# Pathology and immunohistochemistry study of Newcastle disease field case in chicken in Indonesia

**DOI:** 10.14202/vetworld.2017.1066-1071

**Published:** 2017-09-13

**Authors:** Etriwati, Dewi Ratih, Ekowati Handharyani, Surachmi Setiyaningsih

**Affiliations:** 1 Laboratory of Pathology, Faculty of Veterinary Medicine, Syiah Kuala University, Banda Aceh, Indonesia; 2 Department of Clinic Reproduction and Pathology, Faculty of Veterinary Medicine, Bogor Agricultural University, Bogor, Indonesia; 3 Department of Animal Disease and Public Health, Faculty of Veterinary Medicine, Bogor Agricultural University, Bogor, Indonesia

**Keywords:** broiler, domestic chicken, immunohistochemistry, layer, Newcastle disease

## Abstract

**Aim:**

The aim of the study was to examine pathology and the distribution pattern of Newcastle disease virus (NDV) in internal organs of chickens from a field case using immunohistochemical staining.

**Materials and Methods:**

10 groups of broiler, layer, and domestic chicken were collected from necropsy room Division of Pathology, Bogor Agricultural University. These chickens were originated from West Java and collected based on pathologist diagnosis as suspect of Newcastle disease (ND). They were subsequently confirmed positive of ND with real-time-reverse transcription polymerase chain reaction assay. The respiratory, circulatory, digestive, lymphoreticular and central nervous systems were collected for histopathology examination.

**Results:**

The gross pathology and histopathology changes were tracheitis, pneumonia, pericarditis, myocarditis, catarrhal proventriculitis, catarrhal enteritis, typhlitis, perihepatitis, pancreatitis, nephritis interstitial, splenitis, atrophy of Bursa Fabricius, and encephalitis.

**Conclusion:**

The distribution pattern of NDV in internal organs of chickens from a field case in this study is similar with a previous reported pattern in systemic cases of the internal chicken organs. High intensity of immunohistochemistry stain result was detected in trachea, lung, proventriculus, duodenum, cecal tonsil, kidney, and brain.

## Introduction

Newcastle disease (ND) is an important avian disease in Indonesia. ND has spread all over Indonesia and cause massive loss in poultry business. The disease is spreading rapidly, both morbidity and mortality of ND virus (NDV) infection can reach 100% which known to have infected over 200 bird species [1]. The causative agent of the disease is NDV also designated as avian paramyxovirus Type-1, genus avulavirus. NDV is a part of ribonucleic acid virus with the single stranded genome and negative polarity. Based on its virulence, NDV is divided into four groups, which are velogenic viscerotropic (Asian type) characterized by acute lethal infection with intestinal hemorrhage, velogenic neurotropic (America type) characterized by respiration and nerve lesion without intestinal lesion with high mortality, mesogenic characterized by respiratory and nerve lesion with low mortality, and lentogenic characterized by asymptomatic lesion in the intestine [2,3].

The emergence of new genotype variant is estimated from global epizootic and genomic sequence changes in low- and high-virulence NDV indirectly. This hypothesis is that NDV genotype change happened at the same time in various places worldwide [3]. ND outbreak in Indonesia happened first time in Java, island on 1926 [2]. Ever since the outbreak, vaccination program management has been implemented, however until now Indonesia is still an endemic area of ND. Moreover, virus circulation can be detected throughout the year. ND outbreak in 2009-2010 in commercial chicken caused 70-80% mortality [4]. Pathological findings, phylogenetic analysis, amino acid sequence of the F protein cleavage site, and pathogenicity index test results revealed the NDV isolate, designated as NDV/Bali-1/07, to be a novel Indonesia velogenic NDV strain belonging to Group VII [5]. Since 2011 and during 2012 highly related 40 NDV isolates from subgenotype VIIi have been isolated from poultry production facilities and occasionally from pet birds, throughout Indonesia, Pakistan, and Israel [6]. NDV pathogenicity spreading in Indonesia today is a part of virulent strain (mesogenic/velogenic) [4,5,7]. Based on those report, virus strain in Indonesia has been estimated underwent genetic changes. Diagnosis steps that have been established by pathologist in the field in determining the course of the disease are still relying on ND pathognomonic symptoms and reported lesions. Lesions most reported in ND infection is matting of vent feathers, petechiae hemorrhage on the tip of proventriculus papilla, ventriculus, intestine and cecal tonsil, congestion of trachea and lung, and degeneration of ovarian follicles [4,8-10].

In field case, ND lesions are often easily confused with other poultry diseases, therefore to ascertain whether the tissue lesions are caused by NDV infection or not, studies about antigen distribution is critical in observing the existence of NDV within tissue. Immunohistochemistry staining can be used to explain pathogenesis of ND in field cases. In immunohistochemistry staining, an antibody reacting to a specific antigen is used to localize the antigen within affected tissue, which increases the accuracy of a diagnosis as it allows identification of antigens within tissues [11]. This research aimed to determine NDV distribution pattern in chicken internal organ from field cases in West Java, Indonesia.

## Materials and Methods

### Ethical approval

This research has been approved by the Animal Care and Use Committee of Research and Community Services Institution, Bogor Agricultural University (IPB) with approval number: 42-2017 IPB.

### Research procedure

10 field cases from female and male broiler chicken aged 3 and 4 weeks, layer chicken aged 14, 26, and 33 weeks, and female domestic chicken aged 8 and 24 weeks. All chickens were diagnosed as infected by ND by pathologist and real-time-reverse transcription polymerase chain reaction test of all samples concluded to be positive of ND. Histopathology organ samples were trachea, lung, heart, proventriculus, duodenum, cecal tonsil, liver, pancreas, kidney, and brain. All organ samples were cut into 1 cm × 1 cm × 0.5 cm and fixed by neutral buffered formalin 10% solution of at least 24 h before they were then made into paraffin block histopathological section.

### Clinical symptoms

Clinical symptoms were obtained from anamnesis with owner which consists of depression, anorexia, greenish-white diarrhea, torticollis, the number morbidity and mortality, the ras, type, and chicken age.

### Macroscopic observation

Macroscopic changes observed from each organ consist of abnormalities in color, shape, consistency, degeneration and necrosis, circulatory disturbances, inflammation, and exudation.

### Microscopic observation

Tissue section that has been fixed on microscope glass was stained by hematoxylin and eosin (H and E). The histopathological lesion assessment was done descriptively according to the presence of degeneration and necrosis, hemorrhage, congestion, edema, and inflammatory cell infiltration. The severity of each organ was graded with the following criteria: Focal lesion distribution (low severity), multifocal (moderate severity), and diffuse (high severity). Observation was conducted using 100× magnification and 3× view field repetition.

### Immunohistochemistry examination

Tissue section that has been attached on a microscopic slide using poly-L-lysine 1% was deparaffinized, rehydrated with distilled water and flowing with tap water for 5 min, then followed by phosphate buffered saline (PBS) Tween [12]. Antigen retrieval process was conducted using Dako REAL™ target retrieval solution (10×) (Dako, S203130) pH 6.0 inside a microwave in 100°C temperature for 15 min. Tissue section was moved and left for 20 min at room temperature and then washed with PBS Tween for 5 min. Endogenous activity blocking was performed by immersion in H_2_O_2_ 3% at room temperature for 15 min, after which the sections were washed with PBS Tween. Nonspecific protein binding blocking was performed using normal goat serum 10% (Dako, X0907) in room temperature for 30 min and the section was washed again by PBS Tween. Primary antibody rabbit anti-NDV HN protein polyclonal antibody (1:500 in antibody diluent, Dako, S3022) was spilled over the section, incubated in room temperature for 1 h and washed by PBS Tween for 5 min. Sections were then dipped in secondary antibody Dako REAL™ envision™/HRP, rabbit/mouse (ENV) (K5007) for 30 min and washed by PBS Tween for 5 min. The Dako REAL™ DAB+chromogen in Dako REAL™ substrate buffer (K5007) was applied in room temperature for 40 s then washed by flowing tap water for 10 min. The sections were immersed in distilled water three turnover 5 min each. Counterstain used was Mayer’s hematoxylin. The degree immunopositive toward ND in each organ was scored as low (1-10 immunopositive cell toward NDV), moderate (11-20 immunopositive cell toward NDV), and high (more than 20 cells immunopositive toward NDV). Observation was done using 400× magnification with 3 times repetition of view field.

### Statistical analysis

Data obtained from macroscopic and microscopic observation were analyzed descriptively and scored based on the lesion distribution. The immunohistochemistry result was described based on immunopositive location and scored according to the rate of immunopositive cell per field of view.

## Results

### Clinical symptoms

Based on anamnesis it is known that both broiler and layer chicken have been vaccinated; however, there was high morbidity and mortality. Whereas in unvaccinated domestic chicken the morbidity and mortality was 100%. Observed symptoms were decrease appetite, lethargy, weight loss, and greenish-white diarrhea in all chicken examined. In young chicken, symptoms include stunted growth and adult hen exhibit lowering egg production and watery egg albumin. Respiratory disorder was more prominent in young. Neurologic disorders exhibited were paralysis and torticollis.

### Respiratory organ

Gross trachea observation revealed catarrhal tracheitis. Microscopically the trachea showed congestion, hemorrhage, mononuclear inflammatory cell infiltration, and edema at almost every trachea sample. The lungs suffered pneumonia with microscopic lesion of congestion, edema, and mononuclear inflammatory infiltration at the inside and the wall of alveoli. Immunohistochemistry staining of trachea revealed immunopositive reaction at cytoplasm of mucous epithelial cells and cytoplasm of macrophage at submucosal layer. Immunopositive reaction in lungs was found in cytoplasm of parabronchial epithelial cell, pneumocyte, and inflammatory cell of alveoli. Based on the type and age of the chicken, the degree of lesion found in trachea by gross, microscopic, and immunohistochemistry was severe on all chicken.

Lung lesion on all type of chicken grossly showed moderate severity, while microscopic lesion and immunopositive reaction showed high severity. By the age of chicken, in all of the age chicken, lung gross lesions were on moderate severity, whereas microscopic lesions and immunopositive reactions showed high severity in every age.

### Circulatory organ

**Figure-1:**
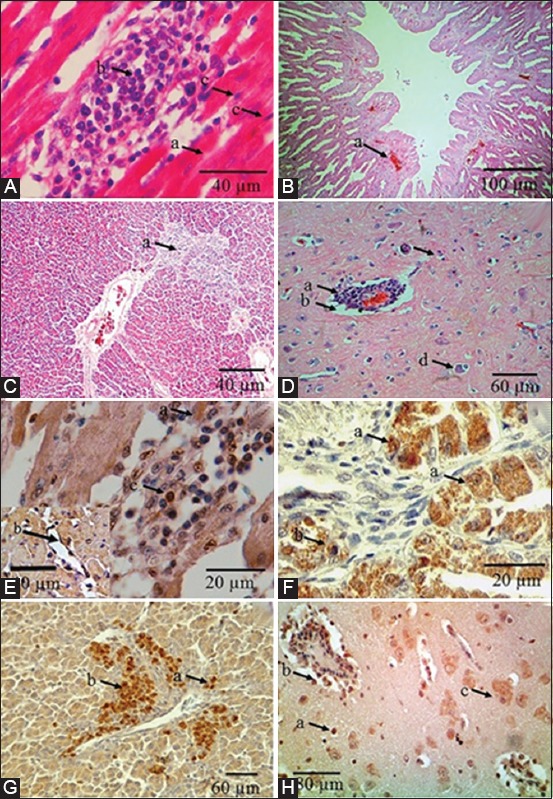
Histopathology changes and distribution of Newcastle disease virus (NDV) in internal organs of chickens from a field case in Indonesia. (A) Heart microscopic observation showed degeneration (a), mononuclear cell infiltration (b), and myocardium necrosis (c). (B) Proventriculus microscopic observation showed hyperemia on glandular epithelial cells of proventriculus glands (a). (C) Liver microscopic observation showed multifocal inflammatory cell infiltration (a). (D) Brain microscopic observation showed hyperemia with perivascular cuffing (a), edema (b), gliosis (c), and satellitosis (d). (E) Heart immunopositive reaction was detected in the cytoplasm of myocardium (a), mononuclear cell infiltration (c), and in vascular endothelial cell (insert) (b). (F) Proventriculus immunopositive reaction was distributed on glandular epithelial cells of proventriculus glands (a), and mononuclear cell infiltration (b). (G) Liver immunopositive reaction was distributed on cytoplasm of hepatocytes (a), and at the macrophage around central vein (b). (H) Brain immunopositive reaction was distributed glial cell (a), mononuclear cells of perivascular cuffing (b), and on neuron cytoplasm (c). Hematoxylin and eosin staining (A, B, C, D), immunohistochemistry staining with rabbit anti-NDV hemagglutinin-neuraminidase protein polyclonal antibody (E, F, G, H).

Heart grossly showed myocardium degeneration. In layer chicken, it was accompanied by pericarditis. Microscopic observation on all heart samples found epicarditis and myocarditis marked by myocardium degeneration, and necrosis, edema, with mononuclear cell infiltration (Figure-1A). Immunopositive reaction in all heart samples was detected in the cytoplasm of myocardium, inflammatory cell, and in vascular endothelial cell (Figure-1E).

Based on the type of chicken, the degree of gross and microscopic lesion of layer chickens were more severe compared to broiler and domestic chicken which have moderate severity. Immunopositive degree was higher in broiler and domestic chicken compared to layer chicken with showed moderate degree. Based on age, macroscopic and microscopic lesion degree is more severe in older chicken compared to young chicken which has moderate severity. However, immunopositive degree was higher in young chicken compared to older chicken.

### Digestive organ

Most proventriculus showed catarrhal proventriculitis and only samples from domestic chicken showed hemorrhagic proventriculitis. Microscopic observation revealed proventriculitis in every sample, marked by desquamation of proventriculus epithelial surface and epithelial gland, hyperemia of the muscularis layer and infiltration of inflammatory cells to submucosal layer of proventriculus glands (Figure-1B). Epithelial mucosal cells, submucosal inflammatory cell, and glandular epithelial cells of proventriculus all showed immunopositive reaction toward NDV (Figure-1F). Based on the type of chicken, proventriculus gross lesion degree in domestic chicken is much severe compared to broiler and layer chicken which only had a moderate degree. Domestic chicken proventriculus showed hemorrhage while broiler and layer chicken only showed swelling with catarrhal exudation. Microscopic and immunohistochemistry observation showed high severity in all chicken type. Based on age difference, macroscopic lesion was spread in moderate degree in all of age; however, microscopic and immunohistochemistry lesion was distributed as high severity in every age.

Duodenum suffered catarrhal enteritis in every sample. Microscopic examination revealed desquamation and necrosis of epithelial mucosal cell, hemorrhage, and inflammatory cell infiltration into submucosal layer. Immunopositive reaction was found mucosal epithelial cells and inflammatory cell at the submucosal layer.

Cecal tonsil showed typhlitis on every sample. Microscopic examination of cecal tonsil revealed that there was necrosis of epithelial mucosal cell, hemorrhage, and infiltration of inflammatory cell into the submucosal layer. Immunopositive reaction was found at mucosal epithelial cells and inflammatory cells at the lymphoid follicle. Macroscopic and microscopic lesion and immunopositive reaction showed high severity on every type and chicken age.

Liver showed degeneration and multifocal necrosis in domestic chicken and broiler chicken, while in layer chicken they were accompanied by perihepatitis. Microscopic examination revealed hemorrhage, necrosis, and multifocal inflammatory cell infiltration (Figure-1C). Immunopositive reaction was found at the macrophage around central vein and the cytoplasm of hepatocytes (Figure-1G).

Pancreas grossly showed multifocal necrotic pancreatitis. While microscopic examination showed multifocal hemorrhage and inflammatory cell accumulation between acinar cells. Immunopositive reaction was found on acinar cells and inflammatory cells. Based on the type of chicken, macroscopic and microscopic lesion and the severity degree in chicken liver and pancreas were more severe in layer chicken compared to moderate degree of broiler and domestic chicken. Higher immunopositive degree of liver and pancreas was found in broiler chicken and domestic chicken, compared with layer chicken which showed moderate degree. Based on age difference, macroscopic and microscopic lesion was more severe in old chicken compared to young, while immunopositive reaction was higher in young compared to older chicken which showed moderate immunopositive.

### Urinary organ

The kidney in every sample grossly had nephritis marked by swelling, fragility with multinecrotic foci. Microscopic examination revealed hemorrhage, tubular epithelial cell necrosis, and inflammatory cell infiltration in the interstitium. Immunopositive reaction was found on tubular epithelial cell, blood vessel endothelial cells, and macrophages within the glomerulus. The degree of macroscopic and microscopic lesion and also immunopositive reaction was severe in every type and every chicken age group.

### Nervous system

The brain grossly suffered encephalitis with microscopic lesion showing hyperemia, perivascular cuffing, edema, gliosis, and satellitosis (Figure-1D). Immunopositive reaction was distributed on neuron cytoplasm, glial cell, and mononuclear cells of perivascular cuffs (Figure-1H). The degree of macroscopic and microscopic lesion and also immunopositive reaction was severe in every type and every chicken age group.

## Discussion

The confirmation of ND suspect chicken diagnosis with immunohistochemical staining in this study proves that the immunohistochemistry method can be used in diagnosing ND accurately, quickly, and more economically than serologically and molecularly identifies viruses. Other advantages include: (1) Can trace the distribution of virus in various organs so it can be used to know the pathogenesis of NDV infection in chickens and (2) safe because of the diluent of the organ that has been fixed with formaldehyde solution so that transmission to sensitive host can be avoided [11].

Respiratory impairment was observable in every chicken because respiratory tract is one of the main gateways of NDV. Virus attaches to the respiratory epithelial cell by utilizing sialic acid on host cell as receptor [13]. The presence of virus on respiratory epithelial cell will induce regional immunity system to produce specific immunoglobulin A antibody which will phagocyte virus. Response observable on the respiratory tract is the presence of congestion and increase of mucous exudate secretion into the trachea, where the body will then attempt to expel virus antigen from respiratory tract, protecting the epithelial surface from virus attachment and invasion [14]. Young bird infected by NDV showed more severe and acute clinical symptoms compared to older bird [2]. Lung lesion is the result of circulatory disturbance caused by viremia and bacterial secondary infection [15]. Immunopositive reaction in both of the respiratory organ matches reports by Nakamura *et al*. [16] that stated trachea, air sac, and lungs are immunopositive toward NDV antigen.

Anemia observed from the carcass, especially at the breast muscle of all samples was associated with the hemorrhage at some internal organ due to NDV replication [17]. Macroscopic and microscopic lesion severity degree in layer chicken was higher compared to broiler chicken and domestic chicken, however, the immunohistochemistry examination showed only moderate immunopositive degree. This may be explained by the presence of pericarditis in layers, which it may be assumed that the more severity erosion may have been caused by the infection of another disease which cause the higher lesion to heart. ND infection is usually accompanied by *Escherichia* *coli* which causes perihepatitis and fibrinous pericarditis, and even fibrinous purulent lesion [18]. NDV distribution in heart tissue was caused by NDV carried by blood into the heart (viremia), marked by immunopositive reaction to blood vessel endothelial cell in heart and other organ. NDV distribution in heart matches previous report by Bwala *et al*. [19] that stated chicken infected by velogenic viscerotropic isolate showed macrophage cell accumulation at the myocardium and immunopositive reaction toward NDV is found at myocardium and mononuclear cells.

Based on observation of clinical symptoms on digestive organ, it is known that the ND infection was of velogenic type. NDV velogenic type infection can cause more severe infection compared to mesogenic or lentogenic NDV. Mesogenic NDV infection can cause macroscopic and microscopic lesion; however, the lesions will spread as much as velogenic virus infection [20]. NDV replication within intestinal lymphoid follicle causes hemorrhage and edema in internal organ due to blood vessel injury [21]. NDV distribution in internal organ is as reported by Nakamura *et al*. [16], Bwala *et al*. [19], that field isolate of NDV antigen was found immunopositive in pancreas, duodenum, proventriculus, and Bursa Fabricius.

Kidney nephritis occurs as the virus invades through respiratory tract then carried by blood circulation to the kidney. Virus replicate inside mucosal epithelial cell of the upper respiratory tract and digestive tract then right after infection virus spread through blood circulation to kidney and bone marrow, causing secondary viremia [13]. NDV distribution in kidney from this research is agreed with previous research by Nakamura *et al*. [16] who found immunopositive reaction toward NDV in kidney tubular epithelial cell.

Clinical symptoms observable originating from the nervous system was caused by nerve cell destruction in the brain from infection and NDV replication. The presence of NDV within brain can cause vascular and neuron damage which will result in an inflammatory response. Inflammatory response begun by macrophrage spread in perivascular cuffing from which then spread to surrounding astrocytes and microgial [22]. NDV distribution in nervous organ system was identical with a previous report [23] which stated that encephalitis was found in infected chicken brain with velogenic viscerotropic isolate and immunopositive reaction to NDV can be located in inflammatory cell and astroglia.

The high incidence of ND in vaccinated and unvaccinated chicken in this research showed that vaccination program currently conducted in the field is not protective enough in preventing disease infection. At present, ND vaccination only protects poultry by decreasing the severity of the disease and lowering mortality, however it cannot prevent NDV replication, especially virulent ND [24]. The lack of protection in vaccines can be affected by various factors. Factors that affect the lack of protection from vaccine today is caused by the presence of genetic difference of strain between virus used in vaccines and the virulent virus strain in the field. The failure of vaccination program in chicken farm (nursery, layer, and meat producer) is caused by the elevating physiological stress in chicken body (internal physiological stress), temperature fluctuation, high humidity, and faulty vaccination program. In addition, the ability of NDV to break through marginal antibody level and has high replication speed in chicken’s body, which may also be among the causes of vaccination program failure [24,25].

## Conclusion

The distribution pattern of 10 field samples used in this research is still the same with previous reported NDV distribution pattern which spread systemically in chicken internal organ. The degree of NDV severity was velogenic viscerotropic, accompanied by velogenic neurothropic. Highest NDV distribution was found on trachea, lungs, proventriculus, duodenum, cecal tonsil, kidney, and brain.

## Authors’ Contributions

EW, DR, and EH executed the experiment, analyzed, and interpretation of data the tissue. EW and SS executed the experiment and analyzed of molecular data. All authors interpreted and critically revised the manuscript for important intellectual contents and approved the final version.
